# Simultaneous treatment of Samter triad and prurigo nodularis with dupilumab

**DOI:** 10.1016/j.jdcr.2021.10.005

**Published:** 2021-10-15

**Authors:** Joshua D. Bloomstein, Jason E. Hawkes

**Affiliations:** Department of Dermatology, University of California Davis, Sacramento, California

**Keywords:** dupilumab, IL-4, IL-13, prurigo nodularis, Samter triad, Th2, AD, atopic dermatitis, CRSwNP, chronic rhinosinusitis with nasal polyposis, IL, interleukin, PN, prurigo nodularis, SNOT, sino-nasal outcome test, ST, Samter triad, Th2, T-helper 2

## Introduction

Samter triad (ST) is characterized by 3 clinical hallmarks: asthma, chronic rhinosinusitis with nasal polyposis (CRSwNP), and a nonallergic hypersensitivity reaction to aspirin. A fourth hallmark, chronic hyperplastic sinusitis, has also been described, making aspirin-exacerbated respiratory disease the preferred name for certain clinicians.^1^ ST is linked to increased T-helper 2 (Th2) activity with increased circulating Interleukin 4 (IL-4), interferon gamma, and leukotrienes.^2^ Prurigo nodularis (PN) is a disease characterized by severely pruritic papules and nodules that result from an intense cycle of itching and scratching. While the exact pathophysiology of PN is not fully elucidated, Th2-derived cytokines (eg, IL-4 and IL-31) have been shown to be overexpressed in classical skin lesions.^3^ There are no approved therapies for PN, and treatment includes topical steroids in combination with reducing picking and scratching behaviors.

Dupilumab is a humanized monoclonal antibody that targets IL-4Rα, resulting in dual inhibition of IL-4 and IL-13.^4^ Dupilumab is approved by the Food and Drug Administration for the treatment of moderate-to-severe atopic dermatitis (AD), CRSwNP, and asthma; however, it is not approved for ST or PN.^5^ The shared role of Th2-related cytokines in the pathogenesis of both ST and PN suggests that dupilumab may be an effective treatment for both conditions as underscored by recent publications.^5^^,^^6^ Here, we report the simultaneous improvement of ST and PN in a patient treated with dupilumab.

## Case report

A 76-year-old man with a history of allergic rhinitis, ST (asthma, CRSwNP, and nonallergic hypersensitivity aspirin), and AD presented to dermatology with generalized pruritus and non healing skin lesions. He reported a history of a recalcitrant methicillin-resistant *Staphylococus aureus* skin infection of the left knee recently treated with trimethoprim-sulfamethoxazole. Clobetasol cream 0.05% applied twice daily for his skin lesions was ineffective, and over-the-counter antihistamines provided little relief for his itching. Of note, the patient also had a history of multiple sinus operations for his CRSwNP, as well as poorly controlled asthma and ineffective desensitization for his aspirin hypersensitivity. Endoscopy 7 months previously showed numerous prominent nasal polyps with a Sino-Nasal Outcome Test (SNOT) score of 8. The patient was already taking daily montelukast for his persistent asthma and allergic rhinitis.

**Fig 1:**
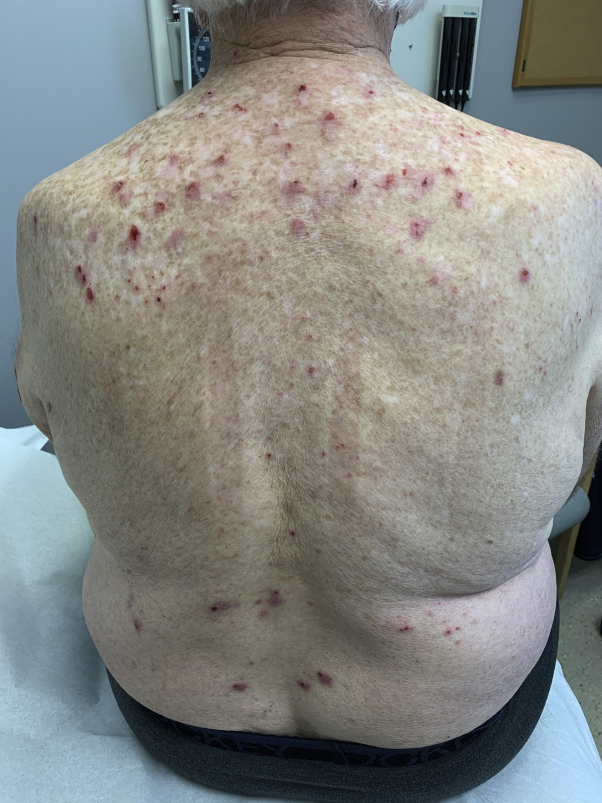
Clinical photo of trunk prior to dupilumab treatment.

**Fig 2:**
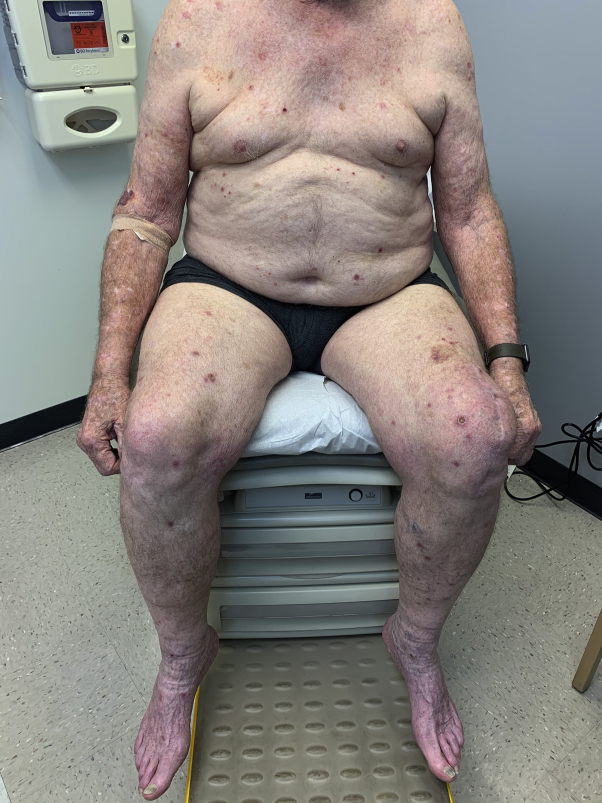
Clinical photo of trunk and extremities prior to dupilumab treatment.

**Fig 3:**
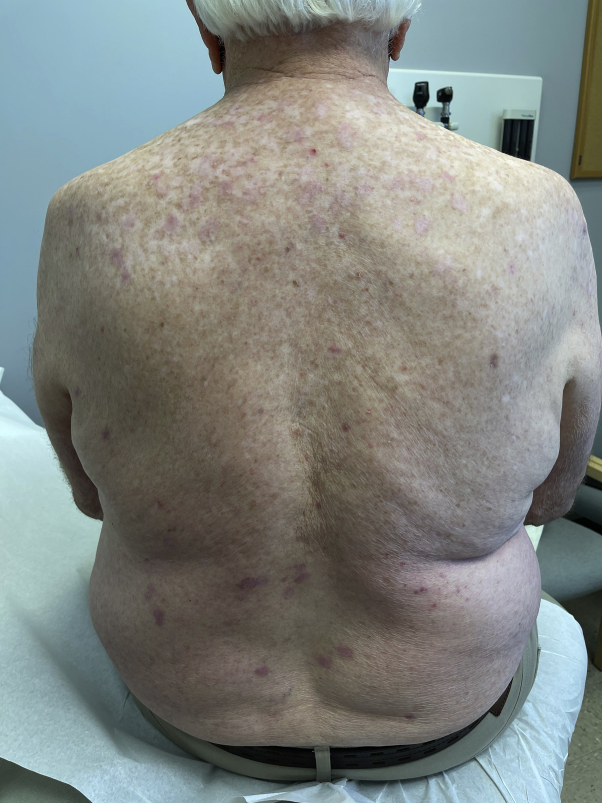
Clinical photo of trunk after 2 months of dupilumab treatment.

Upon conducting physical examination, the patient had multiple erythematous, violaceous papules and nodules with central excoriations on his extremities and trunk sparing the mid-back (Figs 1 and 2). His exam revealed no evidence of active AD. A shave biopsy was performed on a nodule on the right medial thigh and was consistent with the clinical impression of PN. Omalizumab was not tried due to its lack of efficacy for the treatment of non-immunoglobulin E-related conditions, such as the patient's history of AD. Cyclosporine was contraindicated due to the patient's chronic renal insufficiency. Dupilumab (600 mg subcutaneously followed by 300 mg every 2 weeks) was chosen as a preferred treatment given its approved indication for the patient's recalcitrant CRSwNP, asthma, and prior history of AD. The patient reported complete skin clearance within 4 weeks of treatment with dupilumab (Fig 3) with only minor pruritus that was responsive to cetirizine 10 mg. Three months after starting dupilumab, a routine follow-up visit with otolaryngology revealed good nasal airflow with only mild obstruction, reduction in the size of his nasal polyps, and an improved SNOT score of 5. He also indicated that he had resolution of his allergic rhinitis symptoms and a dramatic reduction in his need to use inhaled corticosteroids for asthma-related symptoms.

## Discussion

The standard treatment approach for ST consists of a combination of interventions aimed at clinical symptoms, including inhaled and oral corticosteroids, steroid-sparing asthma therapies, leukotriene-modifying agents, aspirin avoidance or desensitization, and surgical removal of nasal polyps. Monoclonal antibodies (eg, omalizumab and mepolizumab) have also been used and are gaining popularity for treatment of this triad.^7^^,^^8^ However, ST is not primarily driven by increased immunoglobulin E despite its co-occurring allergic diseases such as AD or PN.^2^ Targeted blockade of Th2-derived signals rather than immunoglobulin E for the treatment of ST may represent a novel treatment strategy.

Off-label use of dupilumab is found to be effective for various other atopic and dermatologic conditions, including bullous pemphigoid and PN.^5^ There is only 1 other report describing the use of dupilumab for the treatment of ST. Ten patients were treated for 6 months and showed improvement in both SNOT and asthma measures.^9^ Several case reports and case series have also shown effectiveness of dupilumab for the treatment of PN. One study found that dupilumab treatment led to a reduction of the Numeric Rating Scale Itch Intensity to 0 in 4 patients with recalcitrant PN.^6^ No previous reports have described the simultaneous treatment of ST and PN using dupilumab.

Our report suggests the possibility of a shared underlying immunopathogenesis for ST and PN via increased Th2-derived cytokines given their responsiveness to selective, dual-blockade of IL-4 and IL-13. In a study of 22 individuals with PN, formalin-fixed and paraffin-embedded lesional skin specimens showed increased nuclear localization of phosphorylated signal transducer and activator of transcription 6 in keratinocytes.^10^ Signal transducer and activator of transcription 6 acts as a downstream transcription factor of IL-4 and IL-13 further supporting the role for Th2 cytokines in the pathogenesis of PN. Though ST was originally coined to describe the association of 3 specific clinical entities, the dysregulated Th2 response underlying these clinical hallmarks and their association other atopic conditions via a shared immunopathogenesis may be more important.

Ongoing research uncovering the predominant immune pathways driving the clinical features of related conditions (eg, ST, PN, and AD) may lead to improved management strategies and the subsequent approval of novel treatments for these atopic conditions. This case highlights the importance of considering the immunopathogenesis of the presenting dermatologic condition, as it pertains to similar comorbidities when selecting an appropriate therapy. Ultimately, we hope this case will encourage conversations between dermatologists and other medical specialties, such as otolaryngology or allergy, to consider the use of dupilumab for the simultaneous treatment of PN and other allergic or atopic conditions in shared patients.

## Conflicts of interest

None disclosed.
